# A study of the effect of introduction of JTAS in the emergency room

**DOI:** 10.1002/ams2.266

**Published:** 2017-03-13

**Authors:** Toru Koyama, Takeshi Kashima, Motoyoshi Yamamoto, Kenjiro Ouchi, Takayuki Kotoku, Yuta Mizuno

**Affiliations:** ^1^ Department of Emergency and Critical Care Medicine Aizawa Hospital Nagano Japan

**Keywords:** Afterward inspection, emergency department crowding, US‐originated emergency medicine

## Abstract

**Aim:**

The purpose of this study was to better understand the effects of introducing the Japan Triage and Acuity Scale (JTAS) in the emergency room for walk‐in patients.

**Methods:**

A simple triage was used in Term A (from April 2006 to December 2010, 4 years and 9 months) and the JTAS was introduced in Term B (from January 2011 to September 2015, 4 years and 9 months). The number of patients who had a sudden turn for the worse after arrival in the emergency room and the time between attendance and emergency catheterization (TBAEC) due to acute coronary syndrome were reviewed.

**Results:**

There were 653 patients in Term A and 626 patients in Term B who were finally diagnosed as having serious causes. There was no significant difference in the frequency of a sudden turn for the worse between the two terms. There were 182 patients in Term A and 167 patients in Term B who underwent emergency catheterization due to acute coronary syndrome. When ST elevation was recognized in the first electrocardiogram, the median time between attendance and medical attention during Term B improved significantly, by 4.5 min. However, there was no significant difference in medians for TBAEC. When ST elevation was not recognized, there was no significant difference between the two terms, neither in terms of median time between attendance and medical attention, nor TBAEC.

**Conclusion:**

The data suggests that the effects of introducing the JTAS in the emergency room were restrictive in these two aspects.

## Introduction

After the introduction and spread of US‐originated emergency medicine in Japan, doctors in some specific hospitals are required to treat patients with conditions of varying seriousness, so an adequate triage by nurses has become more important for walk‐in patients in emergency rooms.^1^, ^2^, ^3^ Although there are several triage systems by nurses in emergency rooms,^1^, ^4^ the Japanese Society for Emergency Medicine and the Japanese Association for Emergency Nursing are developing the Japan Triage and Acuity Scale (JTAS, see Appendix) as the standardized triage system in emergency departments.^5^, ^6^ Before the introduction of this kind of systematic triage system, simple triage systems were used in a lot of hospitals. However, there are no studies comparing the effects before and after the introduction of the JTAS.

Among the variety of patients in the emergency center at Aizawa Hospital (Matsumoto, Japan), who visit by themselves with or without somebody's support or who are transferred by ambulance, some patients take a sudden turn for the worse, including cardiopulmonary arrest, after arrival. Even though such serious patients fall into cardiopulmonary arrest just after arrival, problems of triage are not likely to occur as emergency care is immediately started.

When a do not attempt resuscitation (DNAR) order has already been decided by the family, triage problems are not likely to occur either as emergency care is not expected.^7^, ^8^ However, when the condition of walk‐in patients becomes suddenly worse in the waiting room or before and during examination, triage problems possibly arise.

According to a previous report, treatment of patients with serious diseases and myocardial infarction started more quickly after introduction of triage nurses.^1^ So, it is important to assess how long the door‐to‐balloon time has shortened since the introduction of JTAS for patients with acute coronary syndrome, such as ST‐elevated myocardial infarction.^9^, ^10^, ^11^

We previously reported patients who developed cardiopulmonary arrest at the emergency room after arrival.^12^, ^13^ In this report, we study the effects of introducing the JTAS for walk‐in patients in the emergency room using two aspects: (i) the frequency of sudden turn for the worse after arrival among patients who are diagnosed as having serious diseases; (ii) the shortening of the interval to emergency catheterization for patients who are likely to have acute coronary syndrome.

## Methods

From April 2006 to September 2015 (9 years and 6 months), there were 372,908 walk‐in patients who visited the emergency room at Aizawa Hospital. A simple triage was used in Term A (from April 2006 to December 2010, 4 years and 9 months) and the JTAS was introduced in Term B (from January 2011 to September 2015, 4 years and 9 months). Electronic charts of walk‐in patients who were finally diagnosed as having serious diseases were reviewed to select patients with a sudden turn for the worse after arrival. Patients who were referred from other medical institutions after final diagnosis or who had reservation for admission were excluded. According to a previous simple triage, nurses with longer than 3 years of experience undertook triage using 1 or 2 min to pick up patients who had unstable vital signs or various serious symptoms such as loss of consciousness, dyspnea, headache, chest pain, abdominal pain, and back pain. Approximately 80% of patients were checked by triage nurses, and only vital signs were measured by nurses in other patients.

We classified patients as having a serious condition if they died at the emergency room, were transferred to other hospitals for further treatment, or were admitted to the intensive care unit (ICU) or stroke care unit (SCU) at Aizawa Hospital. Among patients who were admitted to the ICU or SCU, those with less serious conditions, for instance, non‐perforated appendicitis, small cerebral infarction/hemorrhage, or acute drug intoxication, who might be able to be treated in a standard ward, were excluded from the serious condition group. We defined a sudden turn for the worse when patients had cardiopulmonary arrest, tracheal intubation, non‐invasive positive pressure ventilation, cardioversion, or sudden loss of consciousness in the emergency room. However, patients with transient loss of consciousness or epilepsy were excluded from sudden turn for the worse.

Of these patients, we investigated age, sex, time between attendance and medical attention (TBAMA), causal diseases of serious condition, time between attendance and sudden turn for the worse, events of sudden turn, causal diseases of sudden turn, and time/location circumstances of sudden turn using electronic charts. We also investigated the existence of ST elevation at first electrocardiogram and entrance time of emergency catheterization in patients who were likely to have acute coronary syndrome. The number of patients who had a sudden turn for the worse in the emergency room and the time between attendance and emergency catheterization (TBAEC) due to acute coronary syndrome were compared between Term A and Term B. The TBAMA was estimated using electronic charts of doctors or nurses when records of starting time of medical attention were lacking. Triage color of JTAS was investigated in Term B. According to the triage method of Aizawa Hospital, patients who needed immediate treatment were regarded as red (emergent level), not as blue (resuscitation level), and transferred to treatment rooms immediately. We did not distinguish green (less‐urgent level) from white (non‐urgent level).

StatView Japanese Version 5.0 (SAS Institute, USA), was used for the statistical analysis. Fisher's exact test and the Mann–Whitney *U*‐test were used for the analysis of the frequency of a sudden turn for the worse and TBAEC, respectively. The results of the analysis were regarded as significant when *P*‐values were less than 0.05.

## Results

**Figure 1:**
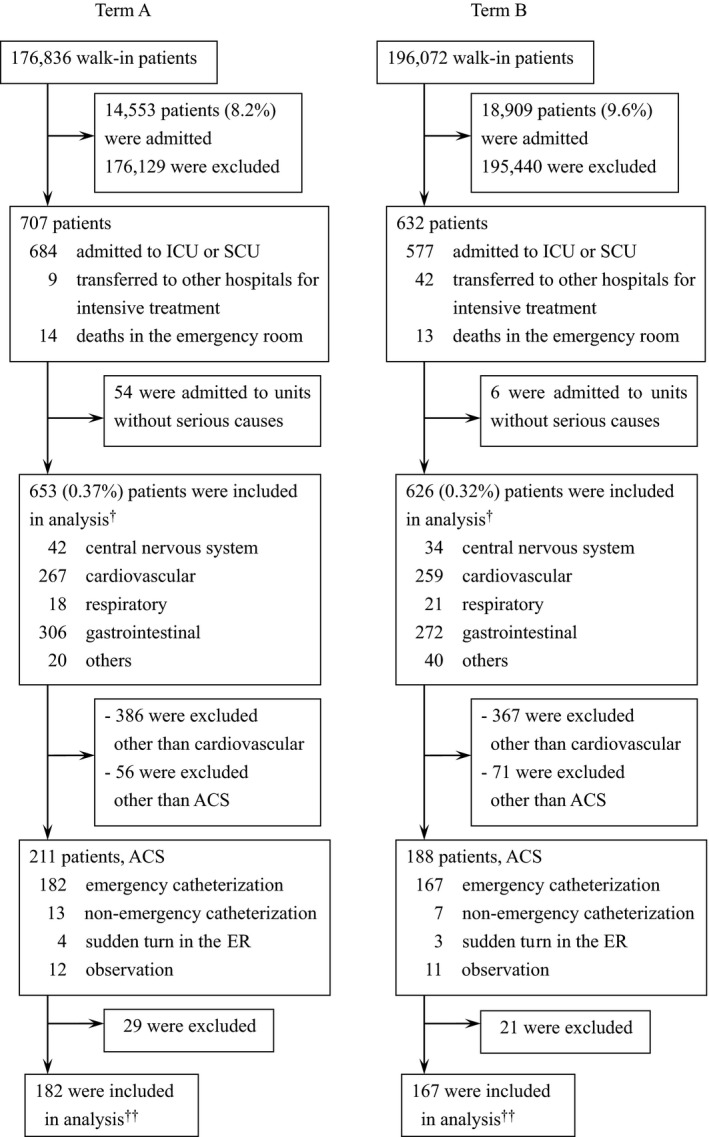
Flow diagram of 1,279 walk‐in patients who were diagnosed as having conditions with serious causes in the emergency room at Aizawa Hospital (Matsumoto, Japan). †Patient data used in Table 1. ‡Patient data used in Table 4. Term A, April 2006–December 2010. Term B, January 2011–September 2015. ACS, acute coronary syndrome; ER, emergency room; ICU, intensive care unit; SCU, stroke care unit.

There were 176,836 walk‐in patients in Term A and 196,072 patients in Term B. After exclusion of patients who went home or were hospitalized to standard wards, 54 patients in Term A and six patients in Term B who were admitted to the ICU or SCU and might have been able to be treated at standard wards were excluded from the serious condition classification. There were 1,279 patients, 653 (0.37%) in Term A and 626 (0.37%) in Term B, who were finally diagnosed as having serious causes (Fig. 1, Table 1). In Term A, there were 384 men, 269 women, 357 patients aged 70 years or older, 296 patients younger than 70 years, and the average TBAMA was 25.2 min. In Term B, there were 390 men, 235 women, 336 patients aged 70 years or older, 290 patients younger than 70 years, and the average TBAMA was 27.1 min. In terms of the causal diseases of serious conditions during Term A, there were 42 patients (6.4%) with central nervous system, 267 (40.9%) with cardiovascular, 18 (2.8%) with respiratory, 306 (46.9%) with gastrointestinal, and 20 (3.1%) with other diseases. In Term B, there were 34 patients (5.4%) with central nervous system, 259 (41.4%) with cardiovascular, 21 (3.4%) with respiratory, 272 (43.4%) with gastrointestinal, and 40 (6.4%) with other diseases. According to triage color, there were 318 patients (50.8%) with red, 170 (27.2%) with yellow, 103 (16.5%) with green, and 33 (5.5%) with unknown in Term B (Table 2).

**Table 1:** Summary of 1,279 walk‐in patients at the emergency room at Aizawa Hospital (Matsumoto, Japan) who were diagnosed as having conditions with serious causes

	Term A	Term B	*P*‐value
	(*n* = 653)	(*n* = 626)	
Sex, *n* (%)			
Male	384 (58.8)	390 (62.3)	0.2012
Female	269 (41.2)	236 (37.7)	0.2012
Age, *n* (%)			
70 years	357 (54.7)	336 (53.7)	0.7206
<70 years	296 (45.3)	290 (46.3)	0.7206
Average time between attendance and medical attention, min	25.2	27.1	
Causes			
Central nervous system, *n* (%)	42 (6.4)	34 (5.4)	0.4493
Subarachnoid hemorrhage	21	9	
Trauma	10	8	
Hemorrhage, infarction	9	12	
Others	2	5	
Cardiovascular, *n* (%)	267 (40.9)	259 (41.4)	0.8600
Symptoms suggestive of ACS	211	188	
Aortic dissection	36	43	
Heart failure, arrhythmia	19	16	
Others	1	12	
Respiratory, *n* (%)	18 (2.8)	21 (3.4)	0.5340
Pneumonia	15	16	
Pulmonary infarction	1	1	
Others	2	4	
Gastrointestinal, *n* (%)	306 (46.9)	272 (43.4)	0.2206
Ileus, herniation	159	120	
Acute appendicitis	57	40	
Perforation	42	54	
Cholelithiasis, cholecystitis	23	28	
Trauma	8	1	
Others	17	29	
Others, *n* (%)	20 (3.1)	40 (6.4)	0.0049
Triage color, *n* (%)			
Red		318 (50.8)	
Yellow		170 (27.2)	
Green		103 (16.5)	
Unknown		35 (5.5)	

Term A, April 2006–December 2010. Term B, January 2011–September 2015. ACS, acute coronary syndrome.

**Table 2:** Summary of patients whose condition took a sudden turn for the worse in the emergency room at Aizawa Hospital (Matsumoto, Japan)

	Term A	Term B	*P*‐value
	(*n* = 653)	(*n* = 626)	
Number of patients with a sudden turn, *n* (%)	30 (4.6%)	36 (5.8%)	0.3776
Average time between attendance and medical attention, min	16.8	21.1	
Average time between attendance and sudden turn, min	156.2	185.5	
Event, *n* (%)			
Cardiopulmonary arrest	16 (53.3)	13 (36.1)	0.2146
Tracheal intubation	10 (33.3)	8 (22.2)	0.4075
NPPV		5 (13.9)	
Cardioversion	1 (3.3)	3 (8.4)	>0.9999
Sudden loss of consciousness	3 (9.9)	7 (19.4)	0.3266
Cause, *n* (%)			
Central nervous system	3 (10)	5 (13.9)	0.7189
Cardiovascular	11 (36.7)	11 (30.6)	0.6121
Respiratory	9 (30)	7 (19.4)	0.3925
Gastrointestinal	5 (16.7)	6 (16.7)	>0.9999
Others	2 (6.7)	7 (19.4)	0.1659
Time/location circumstances, *n* (%)			
In the waiting room	1 (3.3)	4 (11.1)	0.3663
Before and during examination	9 (30)	4 (11.1)	0.0683
After examination	15 (50)	24 (66.7)	0.2126
DNAR order	5 (16.7)	4 (11.1)	0.7209

Term A, April 2006–December 2010. Term B, January 2011–September 2015. DNAR, do not attempt resuscitation; NPPV, non‐invasive positive pressure ventilation.

The number of patients who had a sudden turn for the worse in Term A was 30 (4.6% in 642 patients), the average TBAMA was 16.8 min, and time between attendance and sudden turn was 156.2 min (Table 2). The number of patients who had a sudden turn for the worse in Term B was 36 (5.8% in 626 patients), the average TBAMA was 21.2 min, and time between attendance and sudden turn was 185.2 min. The following events were associated with the sudden turn: 16 patients with cardiopulmonary arrest, 10 with tracheal intubation, one with cardioversion, and three with sudden loss of consciousness in Term A. In Term B, there were 13 patients with cardiopulmonary arrest, eight with tracheal intubation, five with non‐invasive positive pressure ventilation, three with cardioversion, and seven with sudden loss of consciousness. The following causal diseases were associated with sudden turn for the worse: one patient with central nervous system, 11 with cardiovascular, nine with respiratory, five with gastrointestinal, and two with other diseases in Term A. In Term B, there were five patients with central nervous system, 11 with cardiovascular, seven with respiratory, six with gastrointestinal, and seven with other diseases. Regarding the time/location circumstances, there was one patient (3.3%) whose sudden turn for the worse occurred in the waiting room, nine patients (30%) before and during examination, 15 (50%) after examination, and 5 (16.7%) with DNAR in Term A. There were four patients (11.1%) whose sudden turn for the worse occurred in the waiting room, four (11.1%) before and during examination, 24 (66.7%) after examination, and four (11.1%) with DNAR in Term B. There were 10 patients whose sudden turn for the worse occurred in the waiting room or before/during examination in Term A, and eight patients in Term B. Of these patients, there was one patient each in Term A and Term B for which the waiting time was longer than 15 min and the sudden turn for the worse occurred in the waiting room or within 30 min of the start of medical attention. There was no significant difference in the frequency of a sudden turn for the worse between the two terms (Table 3).

**Table 3:** Association between walk‐in patients diagnosed as having conditions with serious causes and patients whose condition took a sudden turn for the worse in the emergency room at Aizawa Hospital (Matsumoto, Japan)

	Term A	Term B	*P*‐value
	(*n* = 653)	(*n* = 626)	
Number of patients with a sudden turn	30	36	0.3776
Number of patients whose condition might have become a problem due to triage^†^	1^‡^	1^§^	>0.9999

Term A, April 2006–December 2010. Term B, January 2011–September 2015. †Waiting time was longer than 15 min and the sudden turn for the worse occurred in the waiting room or within 30 min of the start of medical attention. ‡A 73‐year‐old woman with cardiopulmonary arrest in the waiting room due to aspiration pneumonia. Time between attendance and medical attention (TBAMA), 45 min; time between attendance and event (sudden turn for the worse) (TBAE), 45 min. §A 56‐year‐old man with loss of consciousness in the waiting room due to subarachnoid hemorrhage. TBAMA, 18 min; TBAE, 18 min. *P*‐value, Fisher's exact test.

**Figure 2:**
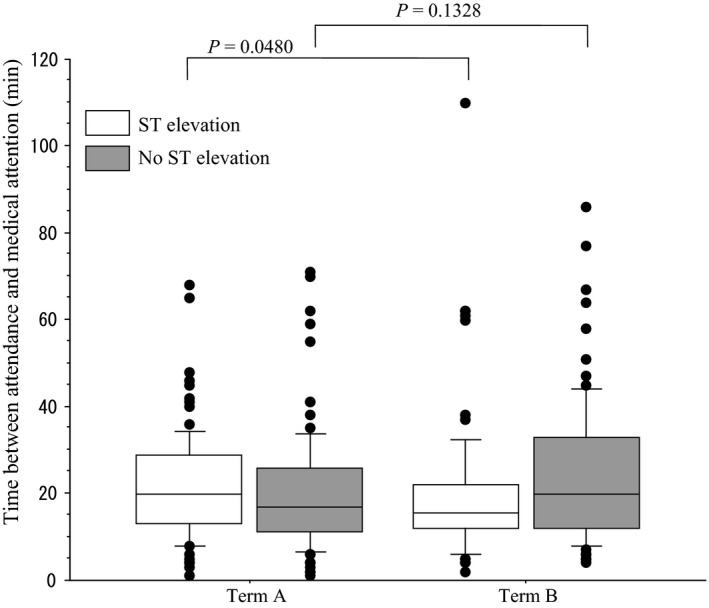
Box and whisker plot of the time between attendance and medical attention in the emergency room at Aizawa Hospital (Matsumoto, Japan). Term A, April 2006–December 2010. Term B, January 2011–September 2015. *P*‐values, Mann–Whitney *U*‐test.

There were 182 patients in Term A and 167 patients in Term B who underwent emergency catheterization (Fig. 1, Table 4). There were 134 men, 48 women, 87 patients aged 70 years or older, and 95 patients younger than 70 years in Term A. There were 136 men, 31 women, 62 patients aged 70 years or older, and 105 patients younger than 70 years in Term B. In Term B, the triage colors were red in 123 (73.6%) patients, yellow in 28 (16.8%), green in 13 (7.8%), and unknown in 3 (1.8%). In terms of causal diseases of emergency catheterization, in Term A, there were 91 patients (50.0%) whose ST elevation was recognized in the first electrocardiogram, 69 (37.9%) whose asynergy was recognized in the ultrasound cardiography, and 22 (12.1%) with other reasons. There were 74 (44.3%), 51 (30.5%), and 42 patients (25.2%), respectively, in Term B. Median TBAMA in Term A was 19 min in total, 20 min when ST elevation was recognized in the first electrocardiogram, and 17 min when ST elevation was not recognized. Median TBAMA in Term B was 19, 15.5, and 20 min, respectively. When ST elevation was recognized in the first electrocardiogram, the median TBAMA of Term B improved significantly, by 4.5 min (Fig. 2, Table 4).

**Table 4:** Summary of 349 walk‐in patients at the emergency room at Aizawa Hospital (Matsumoto, Japan)who were diagnosed as having acute coronary syndrome who needed emergency catheterization

	Term A	Term B	*P*
	(*N* = 182)	(*N* = 167)	
Sex, *n* (%)			
Male	134 (73.6)	136 (81.4)	0.0815
Female	48 (26.4)	31 (18.6)	0.0815
Age, *n* (%)			
≥70 years	87 (47.8)	62 (37.1)	0.0440
<70 years	95 (52.2)	105 (62.9)	0.0440
Triage color, *n* (%)			
Red		123 (73.6)	
Yellow		28 (16.8)	
Green		13 (7.8)	
Unknown		3 (1.8)	
Causes for catheterization, *n* (%)			
ST elevation on ECG	91 (50.0)	74 (44.3)	0.2876
Asynergy on UCG	69 (37.9)	51 (30.5)	0.1474
Others	22 (12.1)	42 (25.2)	0.0016
Median time between attendance and medical attention, min			
Total	19	19	
ST elevation on ECG	20	15.5	
No ST elevation	17	20	
Median time between attendance and catheterization laboratory, min			
Total	102	100	
ST elevation on ECG	74	67.5	
No ST elevation	130	130	
Treatment, *n* (%)			
Stent	135 (74.2)	135 (80.8)	0.1373
Others	47 (25.8)	32 (19.2)	0.1373

Term A, April 2006–December 2010. Term B, January 2011–September 2015. ECG, electrocardiogram; UCG, ultrasound cardiography.

**Figure 3:**
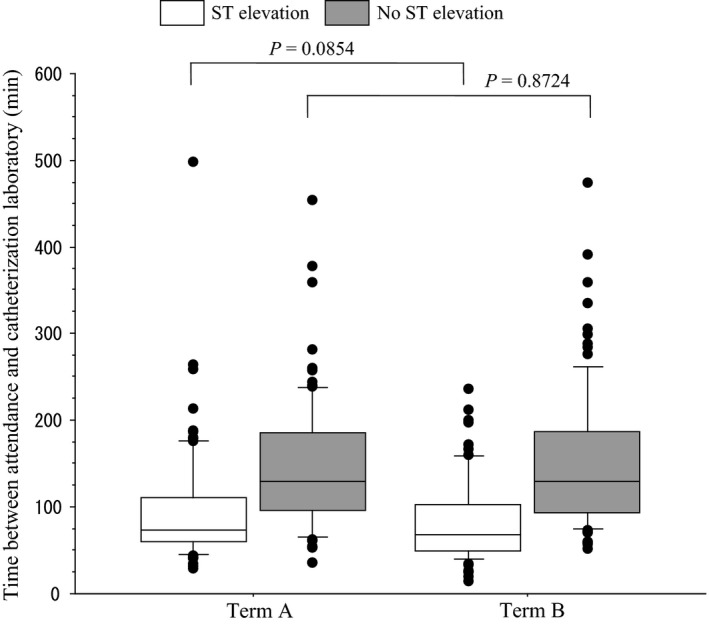
Box and whisker plot of the time between attendance at the emergency room and treatment in the catheterization laboratory at Aizawa Hospital (Matsumoto, Japan). Term A, April 2006–December 2010. Term B, January 2011–September 2015. *P*‐values, Mann–Whitney *U*‐test.

Median TBAEC in Term A was 102 min in total, 74 min when ST elevation was recognized in the first electrocardiogram, and 130 min when ST elevation was not recognized. Median TBAEC in Term B was 100, 67.5, and 130 min, respectively. When ST elevation was recognized in the first electrocardiogram, median TBAEC of Term B improved by 6.5 min, however, there was no significant difference (Fig. 3, Table 4). With regard to treatment at emergency catheterization, there were 135 patients with stent, and 47 patients without stent in Term A. There were 135 and 32 patients, respectively, in Term B.

## Discussion

After the introduction and spread of US‐originated emergency medicine in Japan, too many patients are likely to visit some specific hospitals, so an adequate triage by nurses became especially important for walk‐in patient in emergency rooms.^1^, ^2^, ^3^ Aizawa Hospital is an emergency and critical care center in the Matsumoto area of Japan (background population is approximately 400,000), and approximately 500 beds are available. In addition to full‐time emergency doctors, approximately 30 nurses are assigned at the emergency center, and there are approximately 20 nurses who attended the JTAS provider course. They worked on a day and night shift schedule, and treated a variety of patients who visit by themselves or who were transferred by ambulance.

In the 2014 fiscal year, the number of walk‐in patients was 38,934 (average, 106.7 daily), and the number arriving by ambulance was 7,036 (average, 19.3 daily). The situation of overcrowding changes every day; more than 300 patients may visit in 1 day on consecutive holidays such as new year's holiday or Bon‐festival. Under such situations, the introduction of adequate triage nurses and a systematic triage system such as JTAS is expected to decrease the frequency of sudden turns for the worse in the emergency rooms. According to a report, the percentage agreement between the “quick‐look triage” (carried out using the chief complaint within 30 s) and the Canadian Triage and Acuity Scale was 84.5%, and κ scores were moderately high.^14^ However, after introduction of JTAS, when compared to the previous simple triage system, the decrease in the frequency of sudden turn for the worse was unknown. According to previous reports, TBAEC in cases of myocardial infarction improved after the introduction of systematic triage,^1^ TBAMA improved after the introduction of JTAS,^15^ and TBAMA worsened after the introduction of systematic triage.^16^, ^17^ So, in standard emergency rooms where nursing staff is chronically lacking, there is a doubtful point whether sufficient effect for the same labor has been obtained after the introduction of JTAS.

Although the frequency of over‐ and under‐triage has been discussed many times at afterward inspection,^1^, ^14^, ^15^, ^18^, ^19^ under‐triage is not always directly linked to problems in the emergency rooms. So, we suspected that paying attention to the frequency or time/location circumstances of a sudden turn for the worse in the emergency room would assist in adequately understanding the actual problems of under‐triage.

Current guidelines for the treatment of ST‐segment elevation myocardial infarction recommend a door‐to‐balloon time of 90 min or less for patients undergoing percutaneous coronary intervention;^9^, ^10^, ^11^ therefore, the shortening of the door‐to‐balloon time after introduction of JTAS is noteworthy.

The vague impression that “now is better than before” may be held in many institutions. In this report, we studied the effect of the introduction of JTAS in the emergency room using the frequency of sudden turn for the worse after arrival and TBAEC. There were some patients who fell into the sudden turn for the worse category just after arrival in both Term A and Term B. When we decided that problems of triage possibly exist in patients where the waiting time was longer than 15 min (in reference to objective waiting‐time in case of red, emergent level) and the sudden turn for the worse occurred in the waiting room or occurred within 30 min after the start of medical attention, there was one patient each in Term A and Term B, and there was no significant difference in the frequency of a sudden turn for the worse between the two terms. So we can conclude that the previous simple triage was also effective in this aspect.

In this study, when ST elevation was recognized in the first electrocardiogram in patients with suspected acute myocardial infarction and undergoing emergency catheterization, the median TBAMA in Term B improved significantly, by 4.5 min. However, the data suggest that the effect of introducing the JTAS in the emergency room was restrictive. After announcement of guidelines for the treatment of ST‐segment elevation myocardial infarction, medical examinations for patients with chest pain were likely to start as a priority in order to shorten the door‐to balloon time. However, although the median TBAMA of Term B improved significantly, 4.5 min could not be defined as a dramatic improvement. Such data may provide useful information for other emergency rooms. In cases of walk‐in patients, the time between onset and attendance is likely to be prolonged and chest symptoms are sometimes lacking. So, it is difficult to adequately diagnose patients with possibly acute coronary symptoms, and checking electrocardiograms may be delayed. Using JTAS, it seems that there is a limit to picking up patients who need emergency catheterization from patients who do not have sudden onset of symptoms.^20^, ^21^

In this study, we did not investigate the concrete procedures of JTAS, and only compared the frequency and time/location circumstances of sudden turn for the worse.

We studied 1,279 patients who were finally diagnosed as having conditions with serious causes; among those patients, cases that took a sudden turn for the worse and received subsequent treatment were accumulated. In Aizawa Hospital, there were patients who were intubated and treated in standard wards because of DNAR. In those patients, it may be extremely difficult to identify problem cases with regard to triage, and the method of studying electronic charts of patients who were finally diagnosed as having serious causes and were picked up as problem cases may be reasonable.

In this study, TBAMA was estimated using electronic charts of the first record written by doctors and nurses when the actual starting time of medical attention was lacking. In recent 237 patients, whose actual time records of TBAMA were obtained, the estimated TBAMA was 33.4 min and actual TBAMA was 17.5 min, so the actual TBAMA was shorter than the estimated TBAMA by 15.9 min. In this study, the estimated average TBAMA was 25.2 min in Term A and 27.1 min in Term B. It is probable that medical attention would be actually started within 15 min in patients who were finally diagnosed as having serious causes, so we can say that medical attention will be relatively quickly started at Aizawa Hospital. In order to prevent sudden turns for the worse, we have to not only devise a triage system but also shorten the waiting time. It goes without saying that, to improve TBAEC, we have to not only devise a triage system but also introduce clinical pathways and assign enough medical staff.^22^, ^23^

In this study, we investigated the frequency of a sudden turn for the worse after arrival and TBAEC for patients suspected of acute coronary symptoms, and concluded that “the effect of introduction of JTAS is restrictive” in only those two aspects. However, it is too early to universally conclude the effect of the introduction of JTAS, and further studies, for instance, how much under‐triage is reduced or what kind of clinical improvements are obtained, are expected.

## Conclusion

After their introduction of JTAS in the emergency room, there was no significant difference in the frequency of a sudden turn for the worse. In patients with suspected acute coronary symptoms and subsequent emergency catheterization, when ST elevation was recognized in the first electrocardiogram, median TBAMA improved significantly, by 4.5 min; however, there was no significant difference in median TBAEC. The data suggest that the effects of introducing JTAS in the emergency room were restrictive in these two aspects. In order to disclose the effect of JTAS in the emergency room, further studies, for instance, how much under‐triage is reduced or what kind of clinical improvements are obtained, are necessary.

## Approval of the Research Protocol

This study was approved by the ethical review board at Aizawa Hospital.

## Conflict of Interest

None declared.

## Appendix

The Japanese Society for Emergency Medicine and the Japanese Association for Emergency Nursing are developing the Japan Triage and Acuity Scale (JTAS) as the standardized triage system in emergency departments in Japan. Its fundamental ideas are based on the Canadian Triage and Acuity Scale (CTAS). The prototype of the JTAS was made through translation of the CTAS. Items related to medical conditions commonly seen in Japan, such as heat stroke, were included in the scale.
